# Definite neuroborreliosis with atypical antibody-profiles: a case report

**DOI:** 10.1186/s13256-026-05925-z

**Published:** 2026-03-27

**Authors:** Ingerid Skarstein, Anne Marit Solheim, Hanne Quarsten, Unn Ljøstad, Åse Mygland, Åslaug Rudjord Lorentzen, Randi Eikeland, Harald Reiso, Steffan Daniel Bos, Elling Ulvestad

**Affiliations:** 1 Haukeland University Hostpial, Department of Microbiology, P. O. Box 1400, 5021 Bergen, Norway; 2 Department of Clinical Science, University of Bergen, Bergen, Norway; 3 Department of Neurology, Sørlandet Hospital Trust, Kristiansand, Norway; 4 Department of Clinical Medicine, University of Bergen, Bergen, Norway; 5 Institute of Clinical Medicine, University of Oslo, Oslo, Norway; 6 Department of Microbiology, Sørlandet Hospital Trust, Kristiansand, Norway; 7 Section of Habilitation, Sørlandet Hospital Trust, Kristiansand, Norway; 8 Norwegian National Advisory Unit on Tick-Borne Diseases, Sørlandet Hospital Trust, Kristiansand, Norway; 9 Faculty of Health and Sport Sciences, University of Agder, Grimstad, Norway; 10 Department of Research, Cancer Registry of Norway, Norwegian Institute of Public Health, Oslo, Norway

**Keywords:** Borrelia, Lyme neuroborreliosis, Neuroinfection, Neuroimmunology, Clinical neurology

## Abstract

**Background:**

According to European Academy of Neurology guidelines, a positive *Borrelia burgdorferi* antibody index is required for diagnosing definite Lyme neuroborreliosis. Exceptions to the typical antibody production may be seen in the earlier phases of Lyme neuroborreliosis and in immunocompromised patients with Lyme neuroborreliosis, which can present diagnostic challenges.

**Case presentations:**

We present four Norwegian immunocompetent patients (three male patients aged 52, 61 and 64 years old and one female patient aged 52 years) with neurological symptoms typical of Lyme neuroborreliosis and pleocytosis but a negative or incalculable *Borrelia burgdorferi* antibody index. A positive PCR for *Borrelia burgdorferi* DNA in cerebrospinal fluid confirmed the diagnosis of Lyme neuroborreliosis for all four patients.

**Conclusion:**

Our cases demonstrate that Lyme neuroborreliosis patients with symptom duration for several weeks and a well-functioning immune system can present with atypical antibody profiles. Consequently, we suggest that in cases with pleocytosis and symptoms compatible with Lyme neuroborreliosis but negative *Borrelia burgdorferi* antibody index, one should consider supplementary laboratory testing to confirm the diagnosis.

## Introduction

The diagnosis definite Lyme neuroborreliosis (LNB) requires the presentation of typical symptoms for LNB with other causes excluded, pleocytosis, and a positive *Borrelia burgdorferi *(*Bb*) antibody index [1]. The most common clinical presentation is Bannwarth syndrome—painful meningoradiculitis and/or cranial neuritis (facial palsy), accompanied by subjective complaints such as fatigue, headache, and malaise. Sometimes, however, LNB can present with solely facial palsy or solely subjective symptoms such as newly emerged pain [2, 3]. Affections of the central nervous system, such as myelitis, encephalitis, and cerebral vasculitis, are important but rarer presentations [4, 5].

Patients with newly emerged signs and symptoms compatible with LNB, accompanied by pleocytosis but without detectable *Bb* antibodies in serum or cerebrospinal fluid (CSF) or negative *Bb* antibody index, can pose diagnostic challenges [5, 6]. In such cases, laboratory analyses for the measurement of CSF CXCL13 and CSF *Bb*-DNA are suggested to support the diagnosis. The utilization of alternative antibody assays for detection of distinct immune responses against different *Bb* antigens may also contribute to diagnostic clarification.

The chemokine CXCL13 is a B cell attractant often found at high levels in CSF in LNB, and can be useful as a supplementary marker in early LNB [8, 9]. It is not specific for the diagnosis, however, as it also may present during other causes of neuroinflammation [7]. PCR for the detection of *Bb*-DNA in CSF has a sensitivity of 10–40% and a specificity of nearly 100% [10, 11]. Therefore, PCR is useful when positive, whereas a negative PCR cannot be used to rule out LNB.

We recently conducted a clinical trial in which 121 patients with LNB were randomized to 2- or 6-week treatment with doxycycline. Clinical and laboratory data were collected for a period of 12 months [2, 12]; 83% had positive *Bb* antibody index, and 39 out of 99 (40%) had positive CSF *Bb* PCR [5]. Four patients that displayed negative or incalculable *Bb* antibody indexes prior to treatment were selected for further analyses attempting to confirm the diagnosis.

## Laboratory methods

The laboratory diagnostic work-up was performed according to the procedures at the local inclusion sites during the treatment trial. Therefore, different routine methods for examining *Bb*-specific antibodies were used, the Liaison Borrelia IgG and IgM (Diasorin, Saluggia, Italy) for patient A and the Enzygnost Lyme link VlsE/IgG and IgM (DADE Behring, Marburg, Germany) for patients B, C, and D. For the examination of oligoclonal bands (OCBs), capillary electrophoresis was used for patient A and B, and isoelectric focusing was used for patient C and D [13].

The following tests were not used in the diagnostic work-up but were part of the supplementary tests performed after inclusion in the clinical trial.

CXCL13 was measured by the *recom*Bead CXCL13 (Mikrogen, Neuried, Germany). According to the manufacturer, CXCL13-levels > 300 pg/ml should be considered significantly elevated.

The *recom*Bead Borrelia IgG and IgM-kit (Mikrogen) measures antibody reactivity against 13 recombinant *Bb* antigens with high analytic sensitivity. Several of these antigens are not included in the routine tests.

For *Bb*-PCR, DNA was extracted from 200 µl CSF-cell pellet materials. Two real-time PCR analyses were performed, one for detection of the *OspA* and one for the detection of the 16S rRNA-gene. All DNA samples were tested in duplicates [11].

## Case presentations

### Patient A

Patient A was a 64-year-old Norwegian male individual with a full-time job under treatment with acetylsalicylic acid, atorvastatin, and a beta blocker, who presented with painful thoracal and lumbar radiculopathy, fatigue, neck pain, and serious (defined as having influence on daily life) [12] arthralgia and myalgia over a course of 49 days. He reported having had erythema migrans (EM) within the last 6 months but without receiving any antibiotic treatment. Diagnostic CSF work-up showed 538 white blood cells (WBC)/µL (normal range 0–4 WBC/µL), albumin 1740 mg/L (normal range 70–350 mg/L) and IgG 133 mg/L (normal range 7–66 mg/L). Further CSF findings are shown in Tables 1 and 2. Both *Bb*-IgM and *Bb*-IgG were positive by the Liaison method in the CSF before treatment, but no antibodies were detected in serum, thus making it mathematically impossible to calculate an antibody index ad modum [14, 15]. The CSF collected prior to treatment showed positive PCR for *OspA-* and 16S rRNA-genes and elevated CXCL13-level at >1000 pg/ml. The *recom*Bead supplementary test detected both *Bb*-IgM and *Bb*-IgG reactivity in serum and CSF at inclusion and at 6-months follow-up (Table 2).

**Table 1:** Laboratory data and patient characteristics of the four patients

	Patient A		Patient B		Patient C		Patient D	
	Inclusion	6 months	Inclusion	6 months	Inclusion	6 months	Inclusion	6 months
LNB symptoms and findings	Severe	None	Severe	None	Severe	Mild	Severe	None
Symptom description	Meningoradiculitis, fatigue, joint pain		Facial palsy Fatigue, headache		Radiculopathy, fatigue		Facial palsy, headache, fatigue	
Symptom duration, days	49	–	7	–	21	–	28	–
CSF								
WBC/µl	538	3	8	0	53	NA	74	4
Protein g/L	2.25	0.5	0.36	0.28	0.55	NA	1.07	0.5
OCBs number	2	1	1	1	1	NA	1	0
*Bb*-PCR	Pos	NA	Pos	NA	Pos	NA	Pos	NA
CXCL13 pg/ml	>1000	23	>1000	>1000	430	NA	>1000	448
*Bb* antibody index	NA*	NA*	Neg	Neg	Neg	NA	Neg	Neg

6M, 6 months; LNB, Lyme neuroborreliosis; CSF, cerebrospinal fluid; WBC, white blood cells; OCB, oligoclonal bands; *Bb*, *Borrelia burgdorferi*; NA, not available

^#^Oligoclonal bands restricted to CSF. *An antibody index could not be calculated owing to no detectable antibodies in serum

**Table 2:** Antibody data for routine tests and the supplementary antibody test (*recom*Bead) of the four patients. The *recom*Bead test measures antibodies against multiple recombinant *Bb*-antigens, and the table displays IgM- and IgG-reactivities against these

	Patient A				Patient B				Patient C				Patient D			
	Inclusion		6 months		Inclusion		6 months		Inclusion		6 months		Inclusion		6 months	
Routine tests	IgM	IgG	IgM	IgG	IgM	IgG	IgM	IgG	IgM	IgG	IgM	IgG	IgM	IgG	IgM	IgG
Serum	Neg	Neg	Neg	Neg	Neg	Neg	Neg	Neg	Pos	Pos	Pos	Neg	Pos	Pos	Pos	Pos
CSF	Pos	Pos	Neg	Pos	Neg	Neg	Neg	Neg	Neg	Pos	NA	NA	Neg	Pos	Neg	Pos
*Bb* antibody index	NA*		NA*		Neg		Neg		Neg		NA		Neg		Neg	

*recom***Bead supplementary test**	**IgM**	**IgG**	**IgM**	**IgG**	**IgM**	**IgG**	**IgM**	**IgG**	**IgM**	**IgG**	**IgM**	**IgG**	**IgM**	**IgG**	**IgM**	**IgG**
**Serum**																
p100	+	+	+	+	–	–	–	–	+	+	+	+	+	+	+	+
VlsE	+	+	+	+	–	+	–	+	+	+	+	+	+	+	+	+
OspC	+	+	+	+	+	+	+	+	+	+	+	+	+	+	+	+
p18	–	–	–	–	+	+	+	+	–	–	–	–	–	–	–	–
**CSF**																
p100	–	–	–	–	–	–	–	–	–	–	–	–	–	–	–	–
VlsE	+	+	–	+	–	–	–	–	–	+	–	–	–	–	–	–
OspC	–	–	–	–	–	–	–	–	–	–	–	–	–	–	–	–
p18	–	–	–	+	–	+	–	+	–	–	–	–	–	–	–	–

CSF, cerebrospinal fluid; *Bb*, *Borrelia burgdorferi*; NA, not available; VlsE,variable major protein-like sequence, expressed; OspC, Outer surface protein C

^*^An antibody index could not be calculated owing to no detectable antibodies in serum.

The patient was treated with oral doxycycline for 6 weeks and made a full recovery. At 6-month follow-up, *Bb*-IgG was still positive in CSF but not in serum.

### Patient B

Patient B was a 52-year-old job-seeking Norwegian male individual with no concomitant diseases or medication who presented with severe facial palsy, headache and fatigue over a course of 7 days. He reported a tick bite but no EM within the last 6 months. A diagnostic CSF work-up before treatment showed 8 WBC/µL (normal range 0–4 WBC/µL) and IgG 22 mg/L (normal). Albumin-levels in CSF were not available. Further CSF findings are presented in Tables 1 and 2. Antibodies against *Bb* were not detected in serum or CSF by the Enzygnost method. The CSF collected prior to treatment showed positive PCR for the *OspA*-gene, and elevated CXCL13 at > 1000 pg/ml. The *recom*Bead supplementary test detected *Bb*-IgM and *Bb*-IgG reactivity in serum and *Bb*-IgG reactivity in CSF.

The patient was treated with oral doxycycline for 2 weeks. At 10 weeks after initiation of treatment, he still had mild facial palsy, but at the 6-month follow-up, he was fully recovered. At the 6-month follow-up, the CSF cell count had normalized, but CSF CXCL13 was unchanged, elevated at >1000 pg/ml. Antibodies against *Bb* were not detected in serum or CSF.

### Patient C

Patient C was a 52-year-old Norwegian woman working part time, with a medical history with hip bursitis and migraine, treated with lansoprazole and sumatriptan, who presented with serious lumbar radiculopathy and fatigue over a course of 21 days. She reported a tick bite but no EM within the last 6 months. The CSF diagnostic work-up showed 53 WBC/µl (normal range 0–4 WBC/µL). Levels of albumin and IgG in CSF were not available to us. Further CSF findings are described in Tables 1 and 2. *Bb*-IgG in CSF was detected, as well as *Bb*-IgG and *Bb*-IgM in serum, but the *Bb* antibody index was negative. The CSF collected prior to treatment showed positive PCR for the *OspA* and 16S rRNA-genes and elevated CXCL13 at 430 pg/ml. The *recom*Bead supplementary test detected *Bb*-IgM- and *Bb*-IgG reactivity in serum and *Bb*-IgG reactivity in CSF.

The patient was treated with oral doxycycline for 6 weeks. Although she reported full recovery at 10 weeks follow-up, she reported persistent fatigue and concentration/memory problems related to the LNB at 6- and 12-month follow-ups.

A repeated lumbar puncture was not performed. The *Bb*-IgM in serum remained positive both at 6 and 12 months, but *Bb*-IgG was not detected.

### Patient D

Patient D was a 61-year-old Norwegian man working full time, with rheumatoid arthritis treated with salazypirin, calcigran forte, and 5 mg prednisolone daily, who presented with serious headache, neck pain, fatigue, malaise and a mild facial palsy over a course of 28 days. He did not report tick bite or EM within the last 6 months. The CSF diagnostic work-up prior to treatment showed 74 WBC/µl (normal range 0–4 WBC/µL) and IgG 70 mg/L (normal range 7–66 mg/L). Further CSF-findings are presented in Tables 1 and 2. *Bb*-IgG, but not *Bb*-IgM, was detected in CSF, and both *Bb*-IgG and *Bb*-IgM were detected in serum. The *Bb* antibody index was negative. The CSF collected prior to treatment showed positive PCR for the *OspA-* and 16S rRNA-genes, and elevated CXCL13 at >1000 pg/ml. The *recom*Bead supplementary test detected *Bb*-IgM and *Bb*-IgG reactivities in serum but not in CSF.

The patient was treated with oral doxycycline for 6 weeks and made a full recovery. At the 6-month follow-up, *Bb*-IgG and *Bb*-IgM were detected in serum, and a repeated lumbar puncture showed normalized cell count, but an elevated CXCL13 at 448 pg/ml. *Bb*-IgG was still detected in the CSF, but the *Bb* antibody index was negative.

## Discussion

We describe four patients with LNB with a negative or incalculable *Bb* antibody index where the diagnosis was confirmed by positive PCR for *Bb* DNA in CSF. All four were immunocompetent, with symptom duration of 1, 3, 4 and 7 weeks. They had typical LNB symptoms, pleocytosis, elevated CSF CXCL-13 and serum *Bb*-IgM and *Bb*-IgG reactivities in the supplementary *recom*Bead assay at diagnosis. All responded favorably to antibiotic treatment.

Intrathecally produced *Bb* antibodies in the absence of *Bb* antibodies in serum have been described in the very early phase of LNB, but our patients provide a reminder of caution when using negative serology to rule out LNB even after a longer duration of symptoms [16]. When using the *recom*Bead Borrelia IgG and IgM-kit, we found reactivity against several *Bb* antigens in serum, even when the routine tests did not show detectable antibodies, reminding us that routine tests do not have perfect diagnostic sensitivity.

Patient B had no detectable *Bb* antibodies either in serum or CSF at presentation as measured with the routine test, but also in this patient antibodies were detected by *recom*Bead. This patient had a short symptom duration (7 days) and is thus comparable to previously described cases with undetectable *Bb* antibodies in both serum and CSF in the early phase of LNB [17]. However, patient B illustrates the benefit of supportive tests such as CSF CXCL-13 and CSF *Bb*-PCR in such situations.

Patients C and D had *Bb*-IgM and *Bb*-IgG antibodies in serum and *Bb*-IgG in CSF before treatment but negative *Bb* antibody indexes. The criteria for definite LNB were therefore not fulfilled. When the diagnosis was confirmed by positive CSF PCR, and supported by positive CSF CXCL-13, one could stop looking for other causes and reassure the patient that antibiotics were the drug of choice. Patient C had *Bb*-IgM reactivity in serum for 12 months, confirmed by both antibody assays used in this study. This rules out unspecific reactivity [18]. Prolonged IgM reactivity in LNB is common, and it is not necessarily related to treatment failure [19]. Patient D used low doses of prednisolone, which could have a potential impact on the immune system. This was, however, probably negligible in this case as he had positive serum antibodies and a high level of CSF CXCL13.

## Conclusion

From these four cases, we can learn that LNB should not be ruled out in patients with LNB compatible symptoms and pleocytosis despite undetectable *Bb* antibodies in serum or/and CSF on routine methods. Atypical *Bb* antibody patterns may occur in patients with LNB even if the symptoms have lasted for several weeks and even in the absence of immunosuppression. Testing with CSF CXCL13, CSF *Bb* PCR, and alternative antibody assays can be useful to support the diagnosis.

## Data Availability

Detailed information on the laboratory findings and other data that support the findings are available from the corresponding author upon reasonable request.

## Abbreviations

LNB: Lyme neuroborreliosis
*Bb*: *Borrelia burgdorferi*
CSF: Cerebrospinal fluid
OCB: Oligoclonal bands
EM: Erythema migrans
